# A Numerical Study of Slip System Evolution in Ultra-Thin Stainless Steel Foil

**DOI:** 10.3390/ma12111819

**Published:** 2019-06-05

**Authors:** Zhongkai Ren, Wanwan Fan, Jie Hou, Tao Wang

**Affiliations:** 1 College of Mechanical and Vehicle Engineering, Taiyuan University of Technology, Taiyuan 030024, China; zhongkai_0808@126.com (Z.R.); tyutfww@163.com (W.F.); 2 Analysis and Test Center, Taiyuan University of Science and Technology, Taiyuan 030024, China; houjie@yeah.net

**Keywords:** ultra-thin foil, slip system evolution, tensile process, crystal plasticity, numerical simulation, grain orientation

## Abstract

In order to quantitatively describe the effect of the initial grain orientation on the inhomogeneous deformation of 304 austenitic stainless steel foil during tension, a three-dimensional uniaxial tension model was established, based on the crystal plasticity finite element method (CPFEM) and Voronoi polyhedron theory. A three-dimensional representative volume element (RVE) was used to simulate the slip deformation of 304 stainless steel foil with five typical grain orientations under the same engineering strain. The simulation results show that the number and characteristics of active slip systems and the deformation degree of the grain are different due to the different initial grain orientations. The slip systems preferentially initiate at grain boundaries and cause slip system activity at the interior and free surface of the grain. The Brass, S, and Copper oriented 304 stainless steel foil exhibits a high strain hardening index, which is beneficial to strengthening. However, the Cube and Goss oriented 304 stainless steel foil has a low deformation resistance and is prone to plastic deformation.

## 1. Introduction

Precision stainless steel foil with thickness ranging from 0.01 to 0.1 mm is a high-end steel product, which has been widely used in micro-manufacturing, micro-electromechanical, and other sophisticated micro-products [1]. With the increasing miniaturization of components, higher requirements are put forward for high-quality precision stainless steel, such as good ductility, high dimensional accuracy, and low surface roughness [2,3]. When the foil thickness is reduced to microscale, the anisotropy of the material increases, and the size effects become significant [4,5]. In this case, the grain morphology, size, orientation, and grain boundary have important impacts on the plastic deformation behavior [6,7]. Therefore, the traditional macroscopic plastic deformation theory appears to be no longer applicable to the plastic deformation analysis of the stainless steel foil in microscale. 

The crystal plasticity finite element method (CPFEM) is widely used to homogenize the discrete dislocations and analyze the macroscopic effects of dislocation slip, based on the continuum theory, which reflects the microstructural characteristics of materials [8,9,10,11]. The mechanisms involved in the macro and micro-deformation of non-ferrous metal materials during tensile deformation have been studied by many scholars using CPFEM. Si et al. [12] established a polycrystalline model based on CPFEM and Voronoi polyhedron theory, and studied the effect of grain size on inhomogeneous deformation and grain rotation during the deformation process of polycrystalline aluminum. The results showed that the plastic deformation occurred preferentially at grain boundaries, and the multi-system slip caused local hardening near grain boundaries. Hama and Takuda [13,14] used CPFEM to analyze the hardening and unloading behavior of magnesium alloy strips during tensile deformation. They found that the pyramidal slip system dominated in the loading process, and only the base slip systems were activated during unloading. Ritz and Dawson [15] studied the effect of grain morphology on tensile deformation of aluminum alloys using CPFEM, and pointed out that grain morphology had little effect on the anisotropy of elastic deformation but significantly affected the micro-stress distribution in the grain interior and at the grain boundary. Pi et al. [16] analyzed the effects of polycrystalline model type and tensile strain rate on deformation, necking, and texture evolution of polycrystalline aluminum during the tensile process by CPFEM. Their results showed that the stress increased with the increase in strain rate, and the calculated results by CPFEM were closer to the experimental results.

In order to balance the relationship between the total central processing unit (CPU) calculation time and the model size, the representative volume element (RVE) model with the smallest number of grains was adopted by many scholars to reflect the mechanical behavior of macroscopic samples. Nakamachi et al. [17] proposed a crystalline homogenization algorithm based on multi-scale asymptotic series expansion, established the RVE model for a micro polycrystal structure that could satisfy the periodicity condition of crystal orientation distribution, and verified the accuracy of the RVE model through analytical and statistical examinations. Jigh et al. [18] applied the RVE model to predict the mechanical behavior of metal foams with real microstructure, and studied the effects of different boundary conditions on mechanical properties. Zheng et al. [19] established the RVE model based on CPFEM to predict the influence of structural parameters and relative density of multi-stage honeycomb on its equivalent elastic parameters. The accuracy of the RVE model was verified by comparing with the elastic parameters calculated by the relevant theoretical analytical formula. Kamiński et al. [20] used the iterative and generalized stochastic perturbation technique as a finite element method to determine the effective elasticity tensor for rubber reinforced with the carbon black particles, and applied this to the homogenization problem of the RVE model in such a composite. This study showed some significant differences between numerical and analytical homogenization methods in the context of geometrical uncertainty in the RVE of the composite.

Based on the above literature reviews, it is clear that grain size and morphology as well as strain rate do have an important effect on the plastic deformation behavior of materials. At present, research on the effect of grain orientation on the tensile deformation of 304 austenitic stainless steels, based on the three-dimensional CPFEM model, is still limited. In the present paper, a three-dimensional RVE uniaxial tension model is established based on the CPFEM and the Voronoi polyhedron theory. The effects of initial grain orientation on the inhomogeneous deformation and evolution of slip systems of 304 stainless steel foil during uniaxial tension are analyzed in detail. 

## 2. Crystal Plasticity Model 

The main physical mechanism of plastic deformation of the face-centered cubic (FCC) metal at room temperature is dislocation slip [21]. According to the visco-plasticity criterion proposed by Schmid’s law [22], the relationship between the shear strain rate ${\dot{\gamma }}^{\alpha }$ and the resolved shear stress $\tau^{\alpha }$ of each slip system is established in the form of the exponential function as:

(1) $${\dot{\gamma }}^{\alpha }={\dot{\gamma_{0}}}^{\alpha }f^{\alpha }\left(\frac{\tau^{\alpha }}{\tau_{c}^{\alpha }}\right),\;$$

where the resolved shear stress $\tau^{\alpha }$ is the driving force of the slip motion. The slip system α starts when the resolved shear stress reaches the critical value $\tau_{c}^{\alpha }$.

The material rate sensitivity formula, established by Hutchinson [23], is as follows:

(2) $$f^{\alpha }=\frac{\tau^{\alpha }}{\tau_{c}^{\alpha }}\times {\left|\frac{\tau^{\alpha }}{\tau_{c}^{\alpha }}\right|}^{n-1},\;$$

where ${\dot{\gamma_{0}}}^{\alpha }$ is the reference shear strain rate of slip system α, n is the rate sensitive coefficient, n=0 and n=∞ correspond to viscoelasticity and rate-independent materials, respectively, $\tau^{\alpha }$ is the resolved shear stress of the slip system α, $\tau_{c}^{\alpha }$ represents the dislocation slip resistance or critical resolved shear stress of the slip system α, which is the accumulation of the shear strain of the corresponding slip system on the hardening of the slip system α. $\tau_{c}^{\alpha }$ is expressed by:

(3) $${\dot{\tau_{c}}}^{\alpha }=\sum_{\beta =1}^{N}h_{\alpha \beta }\left|\dot{\gamma^{\beta }}\right|,\;$$

where $\dot{\gamma^{\beta }}$ is the shear strain rate of the slip system β and $h_{\alpha \beta }$ is the latent hardening coefficient.

In the present paper, the hardening model proposed by Bassani and Wu [24,25] was used to describe the interactions between different slip systems with different slip coefficients, which can accurately reflect the slipping process of all three hardening stages of FCC metal. The self-hardening moduli $h_{\alpha \alpha }$ can be expressed as follows:

(4) $$h_{\alpha \alpha }=\left[\left(h_{0}-h_{s}\right)sech^{2}\left(\frac{\left(h_{0}-h_{s}\right)\gamma^{\alpha }}{\tau_{s}-\tau_{0}}+h_{s}\right)\right]\times G\left(\gamma^{\beta };\beta \neq \alpha \right)$$

(5) $$G\left(\gamma^{\beta };\beta \neq \alpha \right)=1+\sum_{\beta =1\beta \neq \alpha }^{N}f_{\alpha \beta }\tanh\left(\frac{\gamma^{\beta }}{\gamma_{0}}\right),\;$$

where $h_{0}$ is the initial hardening modulus; $h_{s}$ is the hardening modulus in the easy slip stage; $\tau_{0}$ is the initial critical resolved shear stress; $\tau_{s}$ is the critical shear stress saturation value; $\gamma_{0}$ is the reference shear strain; and $f_{\alpha \beta }$ is the interaction coefficient between the α and β slip system. The factor depends on the geometric relationship of the two slip systems, which is represented by five constants $a_{i}$(i=1∼5).

The latent hardening moduli $h_{\alpha \beta }$ is given by:

(6) $$h_{\alpha \beta }=qh_{\alpha \alpha },\;$$

where q is the latent hardening parameter.

**Figure 1:**
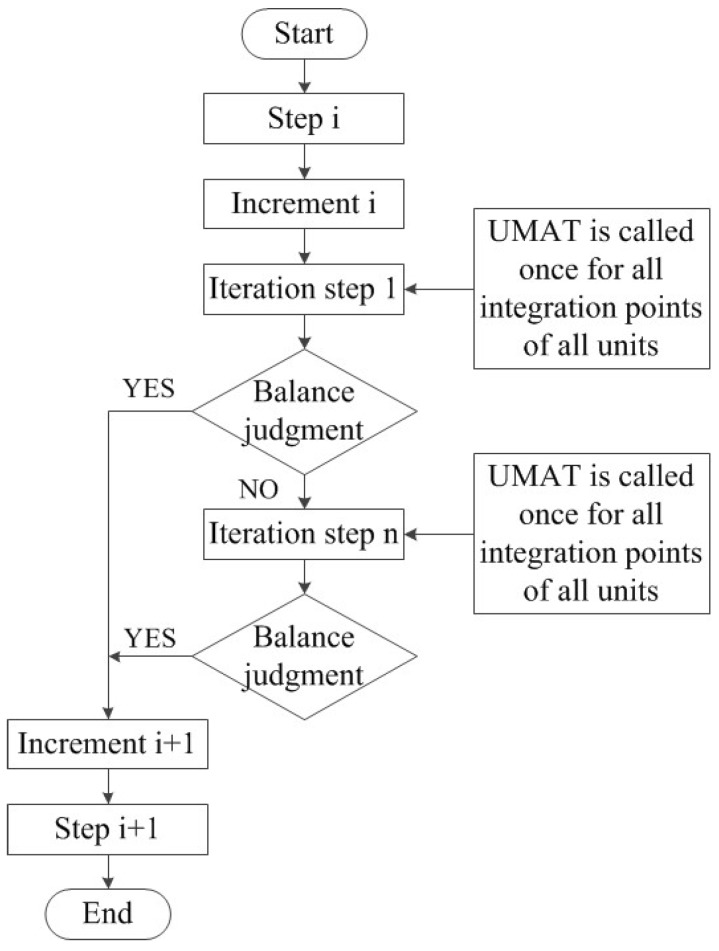
The internal operation flow chart of ABAQUS finite element software.

The above constitutive model was compiled into the user-defined material subroutine (UMAT) of ABAQUS finite element software by Fortran language for subsequent finite element simulation [26], and the combination of crystal plasticity constitutive theory and finite element method was realized. A numerical integration method using implicit differential equations was adopted to solve the rate-dependent crystal plasticity equation. The UMAT used the ABAQUS internal interface to communicate with the main solver. The specific process is shown in Figure 1.

## 3. Finite Element Model for Stainless Steel Foil

X-ray diffraction (XRD) phase analysis of the ultra-thin 304 stainless steel foil (0.05 mm) showed that the main phase constituent was FCC austenite. The elastic constants of single crystal austenite are $C_{11}=209\;\mathrm{GPa}$, $C_{12}=133\;\mathrm{GPa}$, $C_{44}=121\;\mathrm{GPa}$ [27]. Franciosi et al. [28] obtained the interaction coefficients between stainless steel slip systems by hardening experiments, which could be chosen as $a_{1}=0.625$, $a_{2}=0.045$, $a_{3}=0.045$, $a_{4}=0.137$, $a_{5}=0.122$. The remaining unknown parameters could be determined by comparing the simulation results of RVE uniaxial tension with the measured stress–strain curves.

**Figure 2:**
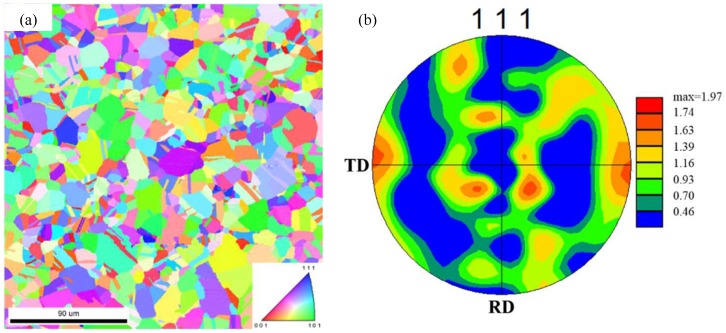
Electron backscatter diffraction (EBSD) mapping of the transverse direction (TD) measured from the rolling direction (RD) of 304 stainless steel foil: (**a**) grain orientation map; (**b**) {111} pole figure.

The average grain size and grain orientation information needed for simulation were obtained by the electron backscatter diffraction (EBSD) tests of the ultra-thin 304 stainless steel foil. EBSD diagrams of 304 stainless steel foil samples measured from the rolling direction (RD) and transverse direction (TD) are shown in Figure 2. The average grain size measured was 15 μm. The maximum density pole of the {111} pole figure was only 1.97 m.r.d (multiple random density), which indicates that the annealed 304 foil had little texture and the grain orientations were almost random. Therefore, the random grain orientations were applied to the polycrystalline model based on the Voronoi polyhedron theory. 

**Figure 3:**
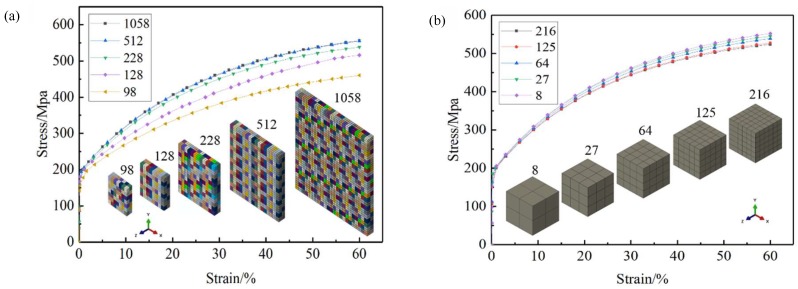
Effect of grain number (**a**) and unit number (**b**) on the representative volume element (RVE) model.

**Figure 4:**
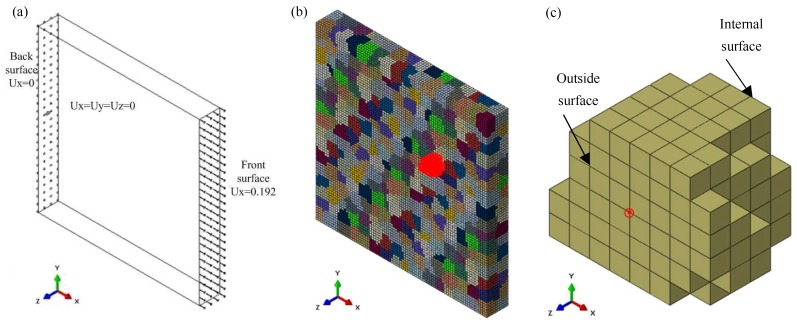
304 stainless steel RVE tensile model: (**a**) boundary conditions in the simulation; (**b**) RVE model; (**c**) G411.

The RVE model was established based on ABAQUS Standard. The effects of the number of grains and the number of elements in the grains on the tensile deformation accuracy of RVE model were analyzed, as shown in Figure 3. The RVE model contained 512 grains with a grain size of about 15 μm, and each grain contained 125 hexahedral elements (C3D8I), with average element dimensions of 3 × 3 × 3 μm^3^ (Figure 4b), which could guarantee the calculation accuracy. Different colors in the model represent different grain orientations, and the aggregates of the same orientation are one grain.

The boundary conditions of the RVE uniaxial tension model are shown in Figure 4a:

(1) The back surface center node was set at X = 0, Y = 0, Z = 0 to avoid the rigid motion of the RVE model as a whole.
(2) The back surface was set at X = 0. There was no constraint in the Y and Z directions, so that plastic deformation could occur in the Y and Z directions.
(3) The front surface was set in a uniform tensile displacement along in the X-direction.

All calculations were run on a workstation (2.6 GHz processor) with 20 multiple processors in parallel. The maximum number of increments was 100,000, to ensure convergence of the RVE model. It took around 5 h to complete one tension test of the RVE with 60% engineering strain.

**Figure 5:**
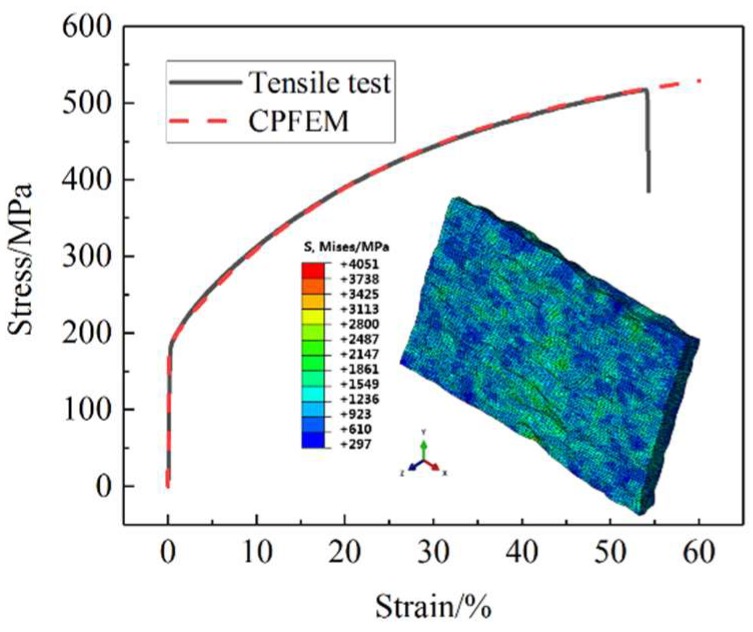
Comparison of the stress–strain curves between RVE simulation and experimental results.

According to the international standard ASTM-E345-16 [29], 0.05 mm thick 304 stainless steel foil tensile specimens were prepared and stretched using an INSTRON-5969 universal tensile testing machine (Instron corporation, Norwood, MA, USA). In the experiment, the stretching rate was 0.03 mm/min. The RVE model established above was subjected to uniaxial tensile simulation to an engineering strain of 60%. The obtained stress–strain curves were compared with the curves measured by tensile tests. The reasonable crystal plastic parameters were determined by optimization (see Table 1), and the comparison results shown in Figure 5 were obtained.

**Figure 6:**
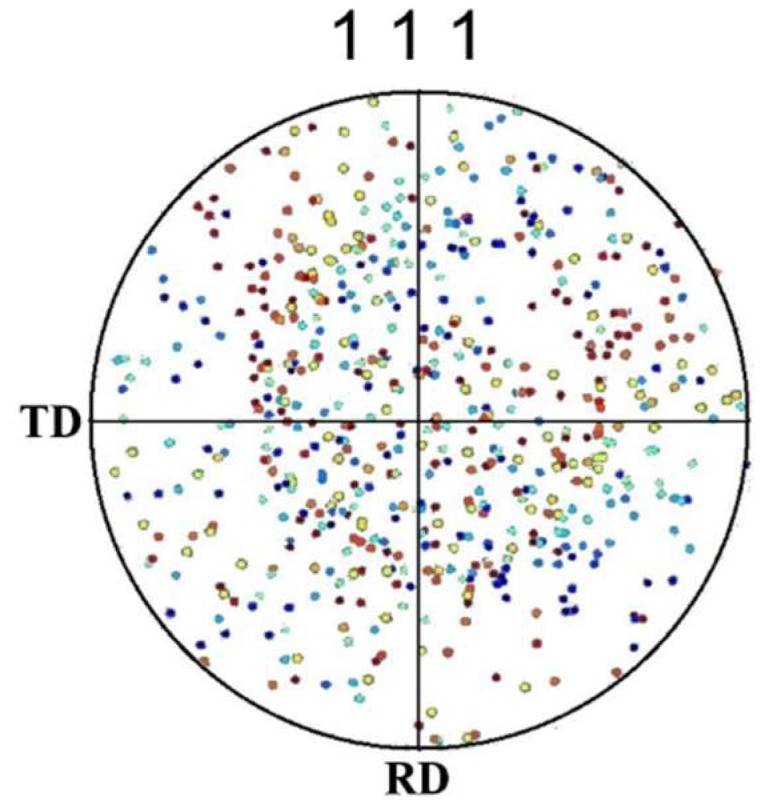
{111} pole figure before RVE deformation with random orientation.

**Figure 7:**
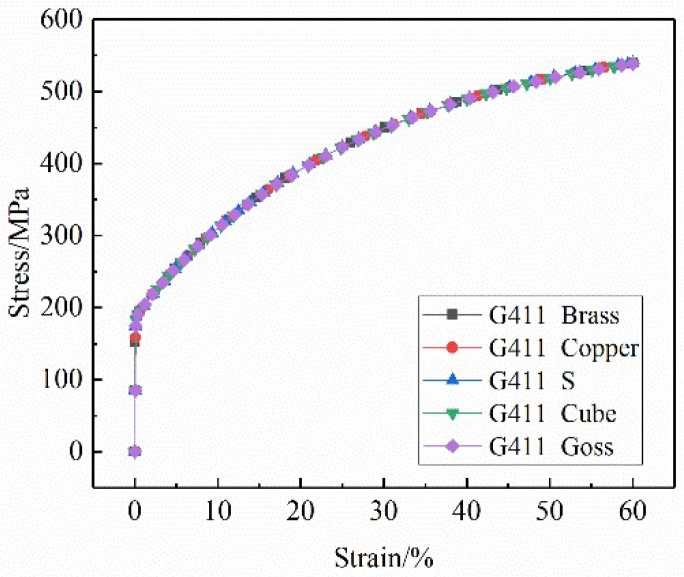
Stress–strain curves of G411 in the RVE model under five different grain orientations.

To study the effect of grain orientation on the motion of slip systems, five typical grain orientations (see Table 2) were assigned to the 411 grain (G411, Figure 4c) in the RVE model, and the other grains were given a random orientation, as shown in Figure 6. Then, the stress–strain curves of uniaxial tension to 60% engineering strain were obtained, as shown in Figure 7. It is clear that the five stress–strain curves coincide completely, indicating that a change of single grain orientation does not affect the mechanical properties of the stainless steel ultra-thin foil. The accuracy of the RVE model established in this paper is verified.

The 304 stainless steel had an FCC crystal structure. The dislocation slip motion occurred on the 12 slip systems {111} <110>, as shown in Table 3. The [100] direction of the crystal was consistent with the X-axis, with [010] and [001] corresponding to the Y-axis and Z-axis, respectively. According to the theory of crystal plasticity, the initiation of the slip system is always controlled by the shear strain rate on the slip system. The negative value of shear strain rate indicates that the slip should be along the opposite direction to the slip system [30].

## 4. Results and Discussion

### 4.1. Microscopic Stress and Strain Distribution of G411

**Figure 8:**
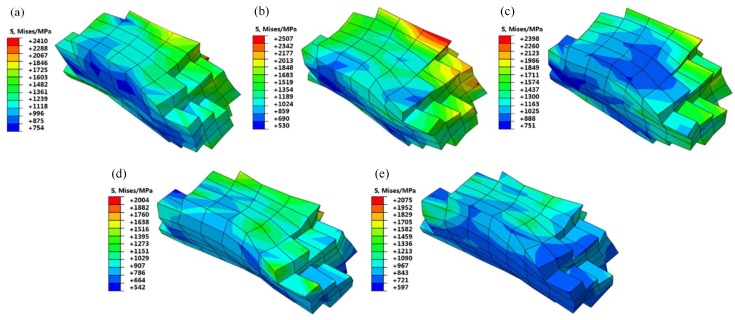
Grain morphology and Mises stress distribution after the G411 deformation: (**a**) Brass; (**b**) Copper; (**c**) S; (**d**) Cube; (**e**) Goss.

Figure 8 shows the stress distribution of G411 under five typical grain orientations for the RVE models with 60% tensile engineering strain. It can be seen that the Brass, Copper, and S oriented grains had an obvious rotation phenomenon. The stress at the internal grain boundary was highly concentrated and the maximum stress value was high, even up to 2507 MPa for the Copper-oriented grain. The grain morphologies of the Cube and Goss oriented grains were similar after tensile deformation, which was mainly elongated along the tensile direction. The stress distribution was relatively uniform and the maximum stress value was small. The maximum stress value of the Cube oriented grain was 2004 MPa. It can be concluded that the different initial grain orientations led to the different degrees of the grain deformation under the same deformation conditions, and the microscopic stress distribution and the maximum stress value differed greatly.

**Figure 9:**
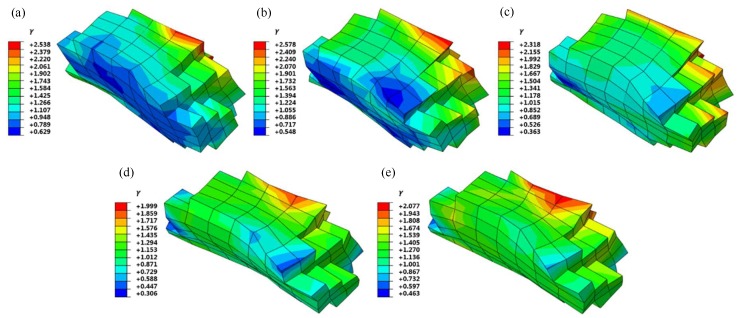
Cumulative shear strain distribution after the G411 deformation: (**a**) Brass; (**b**) Copper; (**c**) S; (**d**) Cube; (**e**) Goss.

Figure 9 shows the accumulative slip distribution of G411 under five typical grain orientations for the RVE models with 60% tensile engineering strain. As can be seen, the cumulative shear strain distributions of five typical oriented grains were not uniform, due to the restraint of adjacent grains, and there were obvious strain gradient characteristics. The strain was mainly concentrated in the boundary region with the internal grain contact. The Cube and Goss oriented grains had large deformations along the X-axis tensile direction. The cumulative shear strain distribution appeared to be more uniform and the cumulative shear strain value was small. The maximum cumulative shear strain value of the Cube oriented grain was 1.999. It can be seen that the initial grain orientation directly affected the cumulative shear strain distribution of the grains.

Considering the deformation coordination of polycrystalline, the grain deformation can be affected by the constraints of adjacent grains. Due to the different initial grain orientations, the order of grain deformation varies with the starting sequence of slip systems in different parts of the grain, so the deformation degree of the grain is different [31]. Compared with the resistance overcome by the slip of the grain internal dislocation, the slip at the grain boundary needs to overcome the resistance caused by the coordination of intergranular deformation, so the stress of polycrystalline plastic deformation is concentrated at the grain boundary [32].

### 4.2. Slip System Evolution of G411 with the Typical Grain Orientations

**Figure 10:**
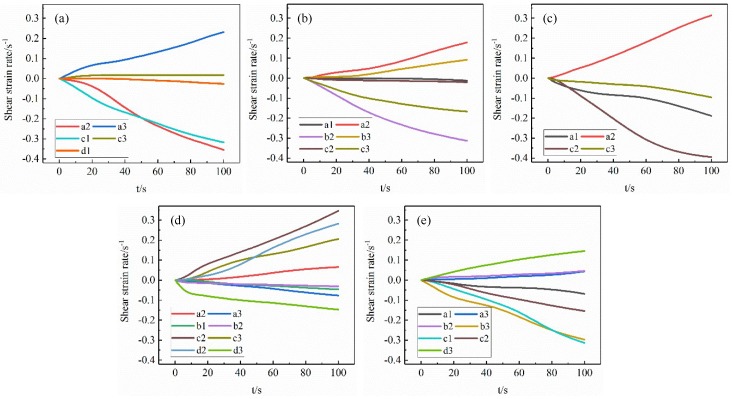
Shear strain rates of a node on the G411: (**a**) Brass; (**b**) Copper; (**c**) S; (**d**) Cube; (**e**) Goss.

In order to investigate the relationship between grain orientation and shear strain rate of the slip systems during tensile deformation, a node on the outside surface of G411, shown in Figure 4c, was selected for analysis, and the results are shown in Figure 10. When the G411 grain orientation was Brass, Copper, or S-type, the number and motion state of the activated slip systems at the node were similar, because of the approximate angles between the three given orientations and the tensile direction. The slip systems a2 and c3 were activated in the above three orientations. Cube and Goss oriented grains had the largest number of activated the slip systems—eight. For the Cube oriented grain, the shear strain rates of slip systems a2, c2, c3, and d2 were large and slipped along their positive directions. The shear strain rates of slip systems a3, b1, b2, and d3 were small and slipped along their opposite directions. For the Goss oriented grain, the slip systems a1, b3, c1, and c2 slid along their opposite directions and the shear strain rates were large, while the slip systems a3, b2, d1, and d3 slid along their positive directions and the shear strain rates were small. It can be concluded that the number and motion state of the activated slip systems at the same position varied greatly because of the different initial grain orientations. The Cube and Goss oriented grains had the largest number of activated the slip systems in the uniaxial tensile stress state, which was beneficial to the occurrence of dislocation slip movement.

**Figure 11:**
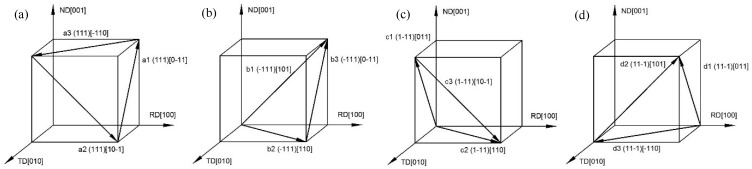
Illustration of {111} planes for initial tensile process: (**a**) (111) slip plane; (**b**) (-111) slip plane; (**c**) (1-11) slip plane; (**d**) (11-1) slip plane.

The starting process of the 12 slip systems of FCC metal during uniaxial tension deformation is shown in Figure 11. When the grain orientation was Cube type, the local coordinate system of the crystal coincided with the global coordinate system of the sample and the angle between grain orientation and tensile force direction was zero. It can be seen that at this time the slip systems of a1, b3, c1 and d1 were perpendicular to the direction of the force. Thus, shear strain did not occur and the shear strain rate was zero. For the Goss grain orientation, the crystal coordinate system did not coincide with the sample coordinate system and the angle between grain orientation and tensile force direction was 90°. At this time, the slip systems a2, b2, c3, d2 were perpendicular to the tensile direction and the shear strain rate was zero. As shown in Figure 10d,e, the other eight slip systems were activated except for the above four slip systems perpendicular to the tensile stress. The shear strain rates of each slip system are different due to the influence of grain morphology and adjacent grains. In summary, the movement of the slip systems in the process of tensile deformation was analyzed by simulation and theory, and the accuracy of the crystal plasticity finite element model was verified.

**Figure 12:**
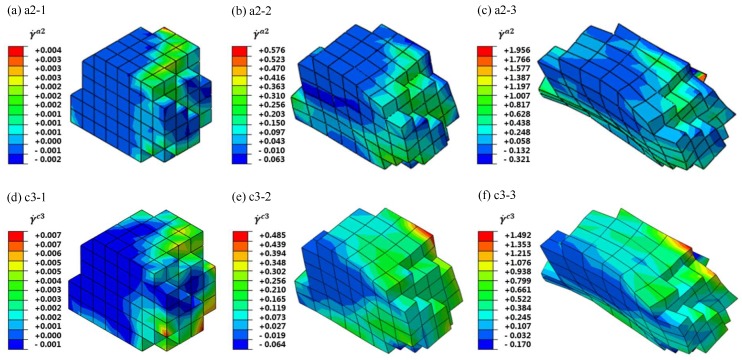
Shear strain rate along active slip system a2 (a2-1, a2-2, a2-3) and c3 (c3-1, c3-2, c3-3) for the G411 of Brass with the tensile time 0.5 s (a2-1, c3-1), 30 s (a2-2, c3-2), 100 s (a2-3, c3-3).

Figure 12 shows the evolution of the shear strain rate of the slip systems a2 and c3 during uniaxial tensile deformation of the Brass oriented G411. It can be observed that the shear strain rate distribution of the slip systems was inhomogeneous. The slip appea red in the opposite direction inside the single crystal grain, which is conducive to the formation of sub-crystallines. The shear strain rate distribution of different slip systems was also various. The a2 and c3 slip systems were activated first at the grain boundaries. With the rotation of the slip surface and direction of the tensile deformation, the internal and free surface slip systems started. The slip strain rate distribution of the slip system a2 varied greatly from −0.321 to 1.956. The outside surface nodes mainly moved along the opposite direction of the slip system a2. The difference of the shear strain rate of the slip system c3 in the whole grain was only 1.662, and the distribution was relatively uniform. It can be seen that the slip systems were activated at the grain boundary first and then caused the movement of the internal and free surface slip systems, and finally formed a slip band in the whole grain. At the same time, the above conclusions can be drawn from the analysis of the shear strain rate evolution process of the other four typical grain orientation slip systems.

Affected by grain orientation, morphology, and polycrystalline deformation compatibility, the number of the activated slip systems and the magnitude of shear strain at different locations are quite different. The motion state of the activated slip systems during plastic deformation is also different. These differences lead to inhomogeneous deformation of the grain and contribute to the stress–strain concentration zone [33].

### 4.3. Stress and Strain Curves of Typical Texture Orientation Dominant Polycrystalline

**Figure 13:**
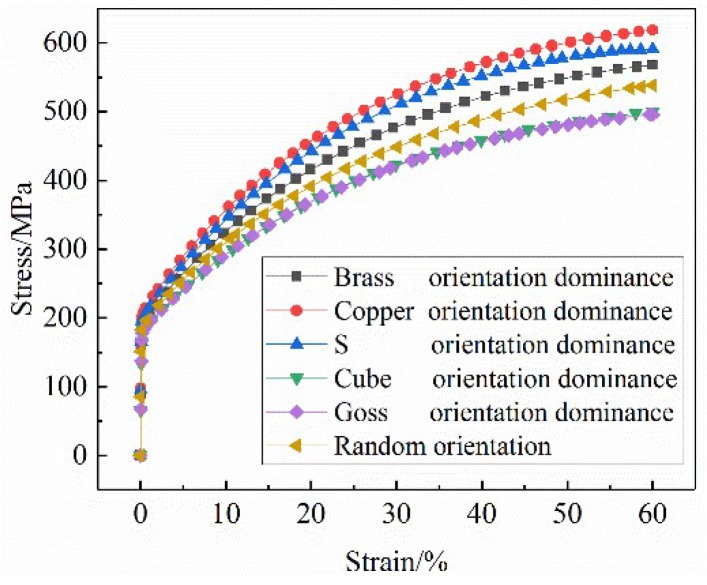
Comparison of stress and strain curves of RVE models with typical texture orientation.

An RVE model with a typical texture orientation was established. The number of grains with each dominant orientation accounted for 50% of the total grains. The simulated stress–strain curves are shown in Figure 13. As can be seen, the yield strength of the RVE model with the dominant texture orientation was approximately 193 MPa, but its deformation resistance varied greatly. In the plastic deformation stage, the stress–strain curves of Brass, S, and Copper oriented dominant models were higher than those of random grain oriented models. The stress–strain curves of Cube oriented and Goss oriented RVE models coincide basically and are significantly lower than those of random oriented RVE models. Under the 60% strain condition, the stress values of the Brass, S, and Copper oriented dominant models are higher than those of random grain oriented model. The maximum stress value of the Copper-type orientation-dominated model is 618 MPa. The stress values of Cube-type and Goss-type orientation-dominated models are approximately 495 MPa, which is lower than that of the random grain orientation model (538 MPa). The results show that Brass, S, and Copper oriented stainless steel materials have large energy storage, large plastic deformation resistance, and a high strain hardening index, which are beneficial to strengthening. Cube-oriented and Goss-oriented stainless steels exhibit low strength and low deformation resistance, and are prone to plastic deformation.

## 5. Conclusions

(1) Based on the crystal plasticity theory and microscopic characterization analysis, a crystal plastic RVE model of 304 stainless steel foil was established. By comparing the tensile stress–strain curves between the RVE simulation and experiment, the calibrated crystalline plastic parameters can accurately reflect the mechanical properties of 304 stainless steel foil. Combined with the simulation and theoretical analysis of the evolution of slip systems during the tensile deformation process, the accuracy of the crystal plastic finite element model was verified.

(2) The crystal plasticity RVE model was used to simulate the effect of typical grain orientation on the inhomogeneous deformation of ultra-thin 304 stainless steel foil during tension. The results show that the numbers of start-up and movement state of the slip systems at the same location are quite different due to the various initial grain orientations, resulting in a great difference in grain deformation degrees and stress–strain distribution. The effect of grain orientation on the plastic deformation of polycrystals is mainly manifested in the mutual constraints and coordination of various grain deformation processes. During the tensile deformation process, the stress distribution and the shear stress with different slip systems are different due to the different initial grain orientation. Therefore, the initiating sequence of the slip systems is different, resulting in different deformation orders and deformation degrees. The grains in favorable orientations slip first, and the grains in unfavorable orientations are difficult to slip.

(3) The change of stress–strain curves in the tensile process of 304 stainless steel foil RVE model with typical orientation was analyzed by CPFEM. The results show that the Brass, S, and Copper oriented dominant models exhibit a high strain hardening index and are beneficial to metal strengthening. However, Cube and Goss oriented dominant models have lower strength and low deformation resistance, so 304 stainless steel foil with these orientations are prone to plastic deformation.

## Figures and Tables

**Table 1:** Constitutive model parameters of ultra-thin 304 stainless steel foil.

n	$\dot{\gamma }$	$h_{0}$	$\tau_{1}$	$\tau_{0}$	$h_{s}$	$\gamma_{0}$	q
60	0.0001	245	30	74	105	0.001	1.0

**Table 2:** Five typical grain orientations of G411.

Number	Grain Orientation	Euler Angles	Miller Indices	Angle to X-Direction
No.1	Brass	(35°, 45°, 90°)	(1 0 1) [$\bar{1}\;\bar{2}\;1$]	110.56°
No.2	Copper	(90°, 35°, 45°)	(1 1 2) [$\bar{1}\;\bar{1}\;1$]	125.26°
No.3	S	(61°, 34°, 64°)	(2 1 3) [$\bar{1}\;\bar{2}\;1$]	116.09°
No.4	Cube	(0°, 0°, 0°)	(0 0 1) [1 0 0]	0°
No.5	Goss	(0°, 45°, 90°)	(1 0 1) [$0\;\bar{1}\;0$]	90°

**Table 3:** Slip systems of face-centered cubic FCC metal.

Slip Plane	Slip Direction	Slip System	Slip Plane	Slip Direction	Slip System
(1 1 1)	[$0\;\bar{1}\;1$]	a1		[0 1 1]	c1
	[$1\;0\;\bar{1}$]	a2	($1\;\bar{1}\;1$)	[1 1 0]	c2
	[$\bar{1}\;1\;0$]	a3		[$1\;0\;\bar{1}$]	c3
($\bar{1}\;1\;1$)	[1 0 1]	b1		[0 1 1]	d1
	[1 1 0]	b2	($1\;1\;\bar{1}$)	[1 0 1]	d2
	[$0\;\bar{1}\;1$]	b3		[$\bar{1}\;1\;0$]	d3
